# Youth friendly reproductive health service utilization and its associated factors among secondary school students, East Belesa district, northwest, Ethiopia, 2022

**DOI:** 10.1186/s12913-023-09152-w

**Published:** 2023-02-22

**Authors:** Menen Tsegaw, Ayenew Kassie, Wallelign Alemnew

**Affiliations:** 1 Department of Public Health, College of Medicine and Health Sciences, Ambo University, Ambo, Ethiopia; 2 Department of Health promotion and Health Behavior, Institute of Public Health, College of Medicine and Health Sciences, University of Gondar, Gondar, Ethiopia

**Keywords:** Youth, Reproductive health, Youth friendly reproductive health services, Youth friendly, Utilization

## Abstract

**Background:**

Youths are people aged between 15 and 24 years. Globally, there were 37.7 million people living with HIV/AIDS, and 90% occur among youths. Despite enormous efforts made in Ethiopia to improve the reproductive health of the youth the utilization is still low. There is no study conducted on YFRHS utilization and associated factors among youths in East Belesa. Therefore, this study is aimed to assess YFRHS utilization and its associated factors among secondary school youths in East Belesa district.

**Objective:**

To assess the prevalence of youth friendly reproductive health service utilization and associated factors among secondary school students in East Belesa district, Ethiopia, 2022.

**Method:**

Institution based cross-sectional study design was used with a total sample size of 347 youths in East Belesa schools from May 23 to June 12, 2022. Stratified simple random sampling was employed. Data were entered using EpiData and analyzed using Stata version 14. Descriptive statistics and Logistic regression were done to describe and identify factors associated with reproductive health services utilization. A P-value of less than 0.05 was considered to declare a level of significance.

**Results:**

A total of 346 students participated in the study with a response rate of 99.8%.the magnitude of youth friendly reproductive health service utilization was 28.9% (24.3, 33.9). Being married (AOR = 0.27, 95%CI: 0.14, 0.52), mothers attended higher education (AOR = 1.40, 95%CI: 1.87, 4.95), availability (AOR = 2.58. 95%CI: 1.29, 5.16) and students who had never discussed about reproductive issues with their families (AOR = 0.18, 95%CI: 0.07, 0.49) were significantly associated with youth friendly service utilization. Therefore, behavior change communication interventions targeted at advancing mothers’ knowledge, encouraging open discussion between parents and children, and enhancing the availability of youth friendly services are important to enhance youth friendly service utilization.

## Introduction

Youths are people aged between the age groups of 15 and 24 years [1–3]. In 2019, the youth population accounted 1.2 billion (16%) of the global population, 211 million (18.3%) of the youths were living in sub-Saharan Africa. In Ethiopia the youth account for one third of the Ethiopian population [1, 4].

Youth Friendly Reproductive Health Services (YFRHS) are those services that are accessible, acceptable and appropriate for the youth including general counseling services about sexuality, voluntary counseling and testing (VCT), treatment and diagnosis of sexually transmitted infections, contraception, condom, perinatal care services, abortion and post abortion care services [2, 4]. Globally, there were 37.7 million people living with Human Immune Virus Acquired Immune Deficiency Syndrome ( HIV/AIDS), and 90% occur among youths [5]. Youth in Sub-Saharan Africa countries are at greater risk of Sexual and Reproductive Health (SRH) problems than those youth from the other parts of the world [6]. The overall utilization of YFRHS in Ethiopia was 42.87% [3].

In Ethiopia, SRH of youth has become a major public health concern [7]. To address the sexual and reproductive health problem of the youth, the Federal Ministry of Health (FMOH) established Youth Friendly Reproductive Health Services (YFRHS) strategy in 2015 [8].

Numerous studies found that sex, maternal education, discussion with families/friends and, educational level, knowledge and attitude were factors associated with intention to use YFRHS [2–4, 6, 9–14]. Despite enormous policies, programs, and efforts that have been made in Ethiopia to improve reproductive health outcomes of the youth, the level of utilization is still very low [3, 15]. To the best of our knowledge there is no study conducted on YFRHS utilization and associated factors among secondary school students in East Belesa. Therefore, this study is aimed to assess YFRHS utilization and its associated factors among secondary school youths in East Belesa district.

## Materials and methods

### Study design and setting

An institution based cross-sectional study was conducted from May 23 to June 12, 2022. The study was conducted in East Belesa district. East Belesa is one of the districts in central Gondar zone, North West Ethiopia, Amhara regional state. It is bordered in the south by South Gondar zone, on the North by Jan Amora, on the West by West Belesa, on the North West by Wegera, and on the East by Wag-Hemra Zone. The district has 22 kebeles. The district is located 720 km far from Addis Ababa (the capital city of Ethiopia). In East Belesa there are four secondary schools including Gohala secondary school (9-12th = 4001), Zuy-Hamusit secondary school (9-12th = 2846), Timen secondary school (9-10th = 693) and Mukatera secondary school (9-10th = 298). There were a total of 7,838 secondary school students attending their education in East Belesa district in those four secondary schools. There is only one youth friendly service clinic in the district.

### Source and study population

All secondary school students in East Belesa district were the source population and all secondary school students who were attending their education in East Belesa district and who were available during data collection were the study population for this study.

### Inclusion and exclusion criteria

All secondary school students who were attending their education in East Belesa district and available during data collection were included in the study. However, Secondary school students who were seriously ill (to the extent of unable to read and write) during data collection period were excluded from the study.

### Sample size determination and sampling procedure

The sample size was determined by using single population proportion formula: $n=(z\alpha /2)2*pq/d$ ^2^. Assumptions: based on following Assumption; Estimated proportion (p) taken from previous study done at Debre Tabor on the utilization of youth friendly reproductive health services among youth (28.8%) [16], Margin of error d = 5%, Confidence level of 95%. The total sample size was: *n* = (1.96)2 (0.288)(0.712) /0.05^2^ = 315. By considering non response rate 10% which is 32, the final sample size was 347. Stratified random sampling technique was used. The total sample was allocated to each grade [9–12] in proportion to their student size. The study participants were selected randomly by using computer-generated random numbers based on a sampling frame prepared by using their identification number (ID) obtained from their respective schools.

### Study variables

#### Dependent variable

Utilization of youth friendly reproductive health services.

#### Independent variables

Socio-demographic variables (age, sex, marital status, residence, religion, education level, living arrangement, monthly pocket money, parental occupation and parental education), sexual experience (yes/no), availability, privacy, convenient time of service delivery (yes/no), communication /discussion with parents (yes/no), distance to health facility (≤ 30 min/>30 min),visited health facility with in the last 12 months (yes/no), media exposure (yes/no), participation in school clubs (yes/no), faced reproductive health problems (yes/no), knowledge (good/poor), and attitude (positive/negative).

### Operational definition

#### Youth Friendly Reproductive Health Service

incorporates general counseling services, Family planning service, VCT, using condom, Treatment of sexually transmitted Infections, and perinatal care (Antenatal Care (ANC), delivery and postnatal care), abortion and post abortion care services.

#### YFRHS utilization

utilization of at least one component of youth friendly reproductive health services.

#### Sexual experience

was assessed by asking the participants “whether they ever had sexual intercourse or not” (yes/no).

#### Knowledge of YFRHS

Four composite score of knowledge items were used to measure the level of knowledge of the respondents regarding youth friendly reproductive health services. For each knowledge, item scores were summed up to get over all knowledge scores, individuals correctly answered the item given a value of “1” and for those answered incorrectly valued ”0”, and then mean and standard deviation were calculated. The mean knowledge score was 7.11 ± 2.21 (*α* = 0.75).

#### Youth reproductive health service utilization

Those respondents who utilize at least one of the following main RH services in the past one year [17].

#### Reproductive health problems

Those respondents who faced at least one of RH problems (unwanted pregnancy, abortion, sexual violence, teenage pregnancy, and Sexually Transmitted Infections (STIs) [17].

#### Attitude towards YFRHS utilization

Attitude (overall evaluation of YFRHS utilization as favorable or unfavorable) was assessed using four items with five point Likert scales. The sum score ranged from 4 to 20 and the higher score indicates favorable attitude towards using YFRHS (α = 0.91).

## Data collection and analysis

Data was collected through a pretested and structured questionnaire after reviewing different relevant literatures [1, 3, 13, 14, 17–23]. Four diploma nurses as data collectors and two B.sc health officers as supervisors were trained for two days. The data was collected by using self-administered structured questionnaire which was prepared by using the local language (Amharic) and translated back to English. Data was entered to EpiData version 4.6 and exported to STATA version 14 for its analysis. The results of the descriptive statistics were summarized by using mean, standard deviation, percentage, frequency tables and graphs. Both binary and multivariable logistic regression analyses were conducted. Those variables which have a p-value of < 0.2 in binary logistic regression analysis were candidate variables for multivariable logistic regression analysis. Moreover, Adjusted Odds ratio (AOR) with 95% confidence interval was used to determine the strength of association between predictor and outcome variables In multivariable logistic regression analysis variables having a p-value < 0.05 with 95% confidence interval were considered as statistically significant.

## Results

### Socio-demographic characteristics of participants

Among 347 students included in the study, 346 participated in the study. with a response rate of 99.8%. The mean age of the participants was 18.7 ± 2.25 years old, and 63.3% of the respondents were within the age group of 15–19 years. Majority of respondents 90.5% were orthodox religion followers. More than half (59.0%) of childhood residents were from a rural area. Majority of respondents were living with their families 83.8% (Table 1 ).

**Table 1:** Socio-demographic characteristics of secondary school students in East Belesa district, North West Ethiopia, 2022 (n = 346)

Variable	Category	Frequency	Percentage
Age (years)	15-19	219	63.3
	20-24	127	36.7
Sex	Male	180	52.0
	Female	166	48.0
Marital status	Single	286	82.7
	Married	60	17.34
Educational level	Grade 9^th^	112	32.3
	Grade 10^th^	114	33.0
	Grade 11^th^	94	27.2
	Grade 12^th^	26	7.5
Religion	Orthodox	313	90.5
	Muslim	33	9.5
Residence	Urban	142	41.0
	Rural	204	59.0
Living arrangement	With families/other relatives	290	83.8
	Alone	56	16.2
Pocket money	No	113	32.7
	Yes	233	67.3
Mothers education	No education	180	52.0
	Primary	86	24.9
	Secondary	43	12.4
	Higher	37	10.7
Fathers education	unable to read and write	117	33.8
	Primary	136	39.3
	Secondary	43	12.4
	Higher	50	14.5
Mothers occupation	Housewife	282	81.5
	Other	64	18.5
Fathers occupation	Farmer	272	78.6
	Government employee	29	8.4
	Private employee	45	13.0
sexual experience	Yes	83	24.1
	No	262	75.9
Availability	Not available	247	71.4
	Available	99	28.6
Privacy	not adequate	262	75.7
	Adequate	84	24.3
convenient time of service delivery	yes	56	16.2
	no	290	83.8
communication with parents	yes	69	19.9
	No	277	80.1
distance to health facility	<30 min	96	27.8
	>30 min	250	72.2
visited health facility with in the last 12 months	Yes	146	42.2
	No	200	57.8
Media exposure	yes	134	38.7
	No	212	61.3
participated in school clubs	Yes	110	31.8
	No	236	68.2
faced RH problems (unwanted pregnancy, STIs)	Yes	36	10.4
	No	310	89.6
Attitude	negative	155	44.80
	Positive	191	55.20

### Knowledge about Youth friendly Reproductive Health Services

Two-thirds 250 (72.3%) of youths have ever heard about YFRHS. The common source of information was health professionals (36.4%) and teachers (23.2%). The most common components of YFRHS by respondents were general counseling service (40.8%) and family planning (31.6%). About (67.2 %) of the respondents had good knowledge on YRHS (Table 2).

**Table 2:** Knowledge about youth reproductive health services among secondary school students in East Belesa district, 2022

Variable	Frequency	Percentage
Have you ever heard about Youth Friendly Reproductive Health services?		
Yes	250	72.3
No	96	27.8
If yes, where do you get the information		
Parent	41	16.4
Friend	29	11.6
Teacher	58	23.2
Mass media	31	12.4
Health professional	91	36.4
Do you know where people get youth friendly reproductive health services?		
Yes	154	61.6
No	96	38.4
Do you know any health facility which gives YFRHS?		
Yes	180	72.0
No	70	28.0
If your answer is yes, which of those facilities do you know?		
Health center	126	70.0
Hospital	32	17.8
School	22	12.2
Which of the following services do you think are being delivered in Reproductive		
General counseling services	102	40.8
FP	79	31.6
VCT	34	13.6
Treatment of STIs	21	8.4
Antenatal care services	14	5.6

### Magnitude of YFRHS utilization

Overall reproductive health utilization was (28.9%) (95% CI: 24.3–33.9). Among the YFRHS components, general counseling services 35 (10.1%), voluntary counseling and testing for HIV 23 (6.7%), STI diagnosis and treatment 17 (4.9%), Family planning 13 (3.8%), Antenatal care services 9 (2.6%) and condom 3 (0.9%) were utilized by school youths in East Belesa district in the last 12 months.

### Factors Associated with utilization of YFRHS

In multiple logistic regression, marital status, mother’s education, availability and discussion with parents were significantly associated with YFRHS utilization.

The odds of YFRHS utilization was 73% lower among married students as compared to single students (AOR = 0.27, 95%CI: 0.14, 0.52). The odds of YFRHS utilization was 1.40 times higher among students having mothers who attended higher education as compared with students having mothers who have no formal education (AOR = 1.40, 95%CI: 1.87, 4.95). The likelihood of YFRHS utilization was 2.58 times higher among students who reported YFRHS are available in their area unlike those students who indicated that YFRHS are not available in the area. (AOR = 2.58. 95%CI:1.29, 5.16). The odds of YFRHS utilization were lowered by 82% among students who had never discussed with reproductive and sexual health issues with their parents/friends as compared as compared with their counterparts (AOR = 0.18, 95%CI: 0.07, 0.49) (Table 3).

**Table 3:** Bivariable and multivariable logistic regressions on factors associated with utilization of youth friendly reproductive health services among secondary school students in East Belesa district in 2022 (*n* = 346)

variable		category	YFRHS utilization			COR		*p*-value	95%CI (AOR)	*P*-value	
			yes		No						
Age		15-19	67		112	1					
		20-24	33		94	1.26		0.362			
Sex		male	50		130	1					
		Female	50		116	0.89		0.631			
marital status	Single		69		217	1			1		
	Married		31		29	0.29		<0.001**	0.27(0.14, 0.52)^a^	<0.001	
Educational level	Grade 9^th^		31		81	1			1		
	Grade 10^th^		42		72	0.66		0.142**	0.96(0.42, 2.18)		0.920
	Grade 11^th^		24		70	1.12		0.35	0.99(0.41, 2.38)		0.977
	Grade 12^th^		3		23	2.93		0.097**	3.35(0.92, 13.5)		0.066
Religion	Orthodox		91	222		1					
	Muslim		9	24		1.09	0.828				
Residence	Urban		34	108		1			1		
	Rural		66	138		0.66	0.091**		0.76(0.44, 1.34)		0.352
Living arrangement	With families/other relatives		84	206		1					
	Alone		16	40		1.02	0.953				
Pocket money	No		32	81		1					
	Yes		68	165		0.96	0.868				
sexual history	Yes		28	55		1			1		
	No		71	191		1.36	0.245**		1.34 (0.72, 2.48)	0.358	
Mothers educational status	No education (ref)		51	129		1			1		
	Primary		22	64		1.15	0.638		1.21(0.61, 2.43)	0.576	
	Secondary		10	33		1.30	0.503		0.65(0.23, 1.78)	0.400	
	Higher		17	20		0.47	0.038**		1.40(1.87, 4.95)^a^	0.038	
mothers occupation	Housewife (ref)		85	197		1					
	Other		15	49		1.41	0.287				
Fathers education	unable to read and write		43	74		1			1		
	Primary		30	106		2.05	0.011**		1.72(0.92, 3.19)		0.088
	Secondary		10	33		1.92	0.111**		1.71(0.63, 4.60)		0.290
	Higher		17	33		1.13	0.734		1.43(0.62, 3.29)		0.398
Fathers occupation	Farmer		7	22		1					
	Government employee		12	33		0.88	0.808				
	Private employee		81	191		0.75	0.527				
Availability	Not available		82	165		1			1		
	Available		18	81		2.24	0.006**		2.58(1.29, 5.16)^a^		0.007
Privacy	not adequate		75	187		1					
	Adequate		25	59		0.95	0.842				
convenient time of service delivery	Yes		15	41		1					
	No		85	205		0.88	0.703				
communication with parents	Yes		8	61		1			1		
	No		92	185		0.26	0.001**		0.18(0.07, 0.49)^a^		0.001
Distance to the health facility	≤30 min		34	62		1			1		
	>30 min		66	184		1.53	0.099**		1.34(0.67, 2.66)		0.406
visited health facility with in the last 12 months	Yes		31	115		1			1		
	No		69	131		0.51	0.008**		0.58(0.29, 1.14)		0.117
Media exposure	Yes		40	94		1					
	No		60	152		1.07	0.757				
Participated in school clubs	Yes		33	77		1					
	No		67	169		1.08	0.758				
faced RH problems (unwanted pregnancy, STIs)	Yes		7	29		1			1		
	No		93	217		0.56	0.191**		0.44(0.14, 1.38)	0.159	
attitude	Negative		42	113		1					
	Positive		58	133		1.28	0.547				
Knowledge	Poor		56	60		1					
	Good		40	94		0.85	0.505				

^a^statistically significant at p-value < 0.05, (ref) reference category

**candidates for multivariable logistic regression

## Discussions

This study is aimed to asses YFRHS utilization and associated factors among secondary school students in East Belesa district 2022. The magnitude of YFRHS utilization was 28.9% (24.3, 33.9). This is in line with studies done in Debre Tabor town, Northwest Ethiopia and Woreta town, South Gondar, North West Ethiopia [1, 16].

This finding is lower than the studies conducted in Amhara region, Harar town, east Ethiopia, Bossat District, Oromia Region, Ethiopia and Bale Zone of Ethiopia [2, 4, 14, 17]. However this finding is higher than the study findings reported in Western Ethiopia, Ambo Town, Oromia Regional State, Ethiopia and Mecha District, Northwest Ethiopia [9, 19, 24]. The possible justification for the discrepancy might be the variation in socio-demographic characteristics, study design and period. Some areas might have better availability and accessibility of YFRHS than others which may affect the utilization of YFRHS among youths and may have a significant role in the observed variation in the magnitude of YFRHS utilization [21, 25].

Marital status was significantly associated with YFRHS utilization. Married students were less likely to use YFRHS than unmarried students. This is in line with the studies done in Gondar city, Ethiopia [26]. This might be due to married students’ decision to use YFRHS may also affected by their spouse/partner. This implies that married students might not be autonomous and make reproductive health decisions alone.

Educational level of mothers was also significantly associated with YFRHS utilization.

Students with mothers who attended higher education were more likely to utilize YFRHS than that of students having mothers who have no formal education [3, 6]. This can be explained that women at higher level of education might have a better access to SRH information and would be more flexible to deal with their children or investigate for any problems their children encounter regarding SRH services use. Maternal education about RH issues influences their children’s ability to freely discuss sexual and reproductive issues, allows them to use RH services and enables children to protect and promote their health and well-being [6].

Availability of youth friendly services was significantly associated with YFRHS utilization. Students who reported YFRHS are available in their area were more likely to use YFRHS unlike those students who declared that YFRHS are not available in the area. This in line with the study done in Kenya and Nigeria [27, 28]. This could be attributed to easy availability of YFRHS may facilitate their motive to use those services.

Discussion with parents about sexual and reproductive issues was significantly associated with YFRHS utilization. Students who had never discussed reproductive and sexual health issues with their parents/friends were less likely to use YFRHS as compared to their counterparts. This is in line with the studies done in Southern Ethiopia, a systematic review and meta-analysis done in Ethiopia [3, 6]. This might be due to the fact that open discussion about SRH issue between family and their children increases awareness and avoids feeling shy and fear of being seen while getting SRH services. Moreover, the discussion creates more opportunities to share SRH information and experience of health-related problems, then the youths would have better knowledge and awareness about SRH services and develop positive attitudes towards YFRHS Which in turn might motivate them to use those services [16, 17].

### Limitations

It is difficult to establish temporal association between YFRHS utilization and its predictors. The study used a self-reporting instrument that has a potential of introducing social desirability bias. This study might be subjected to recall bias since respondents were requested to answer their past experiences.

## Conclusion

The magnitude of youth friendly reproductive health service utilization in East Belesa was low. Marital status, maternal education, availability of services and discussion with families were significantly associated with YFRHS utilization. Therefore, behavior change communication interventions primarily targeted at promoting open discussion between youth and families, enhancing mothers’ knowledge, enhancing the availability of youth friendly services, and school reproductive health clubs especially for those married youths are important to enhance youth friendly service utilization rate. Hence, East Belesa district health offices, East Belesa education offices and other organizations working in the area of youths reproductive health services are recommended to give awareness on youth friendly service utilization for youth’s mothers through provision of training in collaboration with East Belesa town administration.

## Abbreviations

AIDS: Acquired Immune Deficiency Virus
AOR: Adjusted Odds Ratio
CI: Confidence Interval
HIV: Human Immunodeficiency Virus
VCT: Voluntary Counseling and Testing
WHO: World Health Organization
YFRHS: Youth Friendly Reproductive Health Services
